# The Global Contributions of Working Equids to Sustainable Agriculture and Livelihoods in Agenda 2030

**DOI:** 10.1007/s10393-022-01613-8

**Published:** 2022-09-01

**Authors:** D. C. Grace, O. Diall, K. Saville, D. Warboys, P. Ward, I. Wild, B. D. Perry

**Affiliations:** 1 Natural Resources Institute, Central Avenue, University of Greenwich, Chatham Maritime, ME4 4TB Kent UK; 2 Animal and Human Health Program, International Livestock Research Institute, Box 30709, Nairobi, Kenya; 3 Independent Consultant, Bamako, Mali; 4 Brooke–Action for Working Horses and Donkeys, 52-56 Leadenhall Street, London, EC3A 2BJ UK; 5 World Horse Welfare, Anne Colvin House, Snetterton, NR16 2LR Norwich UK; 6 Nuffield College of Clinical Medicine, University of Oxford, Oxford, OX1 1NF UK; 7 College of Medicine and Veterinary Medicine, University of Edinburgh, South Bridge, Edinburgh, EH8 9YL UK

**Keywords:** Working equids, sustainable development goals, agriculture, livelihoods

## Abstract

Small farmers produce most food in low- and middle-income countries and most small farmers rely on directly or indirectly working equids (WE). The lack of methods and metrics for assessing the role of WE hampers realisation of WE contributions. Based on literature review and a survey of WE welfare experts, we propose a framework for optimising WE potential based on two axes of sustainable development goals (SDGs) and value chains. WE contribute especially to earning and sparing income (largely in food production) (SDG 1), but also have roles in accessing health and hygiene services and products (SDG 3 and 5), providing edible products (SDG 2), and benefiting women (SDG 6), with lesser contributions to other SDGs, notably climate action (SDG 13). Experts identified barriers to appropriate appreciation of WE contributions, in order to target actions to overcome them. They found WE are neglected because they belong to farmers who are themselves neglected; because information on WE is inadequate; and, because the unique nature and roles of WE means systems, policies, investors, markets and service providers struggle to cater for them. Harnessing WE to optimally contribute to sustainable development will require generating better evidence on their contributions to SDGs, ensuring better integration into ongoing efforts to attain SDGs, and building the WE capacity among development actors.

## Introduction

The roles played by working equids (WE), comprising horses, donkeys, mules, and hinnies, in supporting agriculture and the livelihoods of smallholder farmers, entrepreneurs, pastoralists, and rural communities in low- and middle-income countries (LMICs) has long been noted (Arriaga-Jordán et al. 2007; van Dijk et al. 2014; Pritchard et al. 2018; Brooke, 2019b). However, their contributions to global agriculture and to the United Nations’ Agenda 2030 Sustainable Development Goals (SDGs) are not sufficiently recognised (Perry 2017), as witnessed by their infrequent inclusion in development and poverty reduction programmes, the fact that they are not categorized as “livestock” by many national livestock ministries (Babayani 2019), and the under-estimation of their contributions to food, nutrition, and livelihood security. Only after extensive lobbying by equid NGOs in 2016 did the United Nations formally recognise WE as livestock and their role in One Health (Horsetalk 2016). WE are an “invisible” or “Cinderella” sector. The few authors who have explored the contributions of WE to poor communities, repeatedly emphasize that data on their small- and large-scale socio-economic contributions are grossly inadequate (Pritchard 2014; Valette 2015). Furthermore, the available data generally appear in the grey literature and are therefore largely unavailable to evidence-aggregators and policymakers. Collecting evidence about the roles of WE in reducing hunger and poverty, empowering marginalised and excluded people (including women) and increasing environmental sustainability and climate resilience is key to elevating their status and the inclusion of equid welfare in national and international policy agendas. This paper attempts to fill this gap by using an integrative review process to combine evidence from the literature on the contribution of WE to SDGs with the expert knowledge of WE professionals to identify barriers to optimising the contributions of WE to SDGs and recommendations for overcoming these barriers.

## Methods

We first conducted a scoping literature review to explore the relation between WE in LMICs and SDGs. Given that this topic has not previously been comprehensively reviewed, and the complexity and heterogeneity of sources, a scoping review was appropriate (Peters et al. 2015). Having confirmed the important contributions of WE to SDGs we sought to identify the barriers to greater understanding and leverage of these contributions in order to develop recommendations for optimising WE contributions. This drew on comments made by WE welfare professionals in different regions of the world, elicited by the authors and including their own experiences, as well as citations the experts provided. As this was interview-based as well as literature-based, it can be considered an “expert integrative scholarly” review. Integrative reviews go beyond the traditional boundaries of systematic reviewing by using experts as sources of evidence, thus increasing the validity of the review through an iterative and interpretivist appraisal (Austen et al. 2016). This approach was chosen because the literature on WE in LMICs is both scarce and slanted undermining the utility of conventional review (Alonso et al. 2016). First, the initial concept of the integrative review was developed by the lead author who then used personal contacts to identify lead experts with backgrounds in research, NGOs, development, and public services with extensive experience in Latin America, Africa and Asia to co-author the study. After briefing on the objectives of the study, an email with open questions was sent to an international cohort of experts from World Horse Welfare. Responses were synthesised by the paper authors and illustrative citations extracted.

### The Global Contributions of WE to the Sustainable Development Goals

According to World Bank data, 80% of the 770 million people living in extreme poverty are located in rural areas, and most of them work in agriculture (The World Bank 2014). These small-scale farmers produce 80% of the developing world's food (Ricciardi et al. 2018), and many rely on WE as key components of their production system. Not atypical is the Kenyan woman who maintained, “Farming is made possible by donkeys. All household animals rely on donkeys which are the ones carrying and bringing feed and water for cows, chickens, sheep and goats” (Valette, 2014).

Acknowledging that WE work is not enough. To better understand the contributions of WE, more granularity is needed on the functions they perform. Standard categorization schemata often do not even distinguish between working and leisure animals, let alone untangle the various and different roles of WE in different farming systems. A major constraint is that, with some exceptions, WE do not have a role in the easily monetised productions of milk, fibre, and meat due to a lack of organized markets for these products. While WE contributions are often summarised under traction and transport, this fails to capture their diversity and depth. What cannot be measured, will not be managed. We propose a more functional characterisation based on the products for which traction and transport are needed which would allow quantification of the contributions made by the product to food and market requirements. Given the diverse and dynamic demands of meeting global nutritional, construction, and livelihood needs, this approach may allow the role of WE to be more comprehensively assessed, including ecosystem, socioeconomic and sustainability implications, and hence foster greater support of WE in development.

Our commodity or more broadly goods- and services-based framework captures the use and livelihood benefits of owning an equid, and we illustrate with examples below of WE’ contributions to the SDGs.

In 2015, the United Nations General Assembly adopted a new development agenda: “Transforming our world: the 2030 agenda for sustainable development”(United Nations 2015). The International Coalition for Working Equids (ICWE) identified eight goals to which they believe WE can contribute (SDGs 1–6, 8, 12), as well as the cross-cutting SDG 17 on partnerships to attain goals (United Nations 2020). Similarly, the Global Agenda for Sustainable Livestock (GASL 2022), a multi-stakeholder partnership, considered eight SDGs especially relevant to livestock (SDGs 1, 2, 3, 5, 8, 12, 13, 15) (Food and Agriculture Organization 2016). Here, we include a review of equid contributions to SDGs 1, 2, 3, 5 and 6, with additional information available in the policy briefs mentioned above. Interestingly, there has been a proposal to create an 18th UN goal on animal concerns by Visseren-Hamakers (2020), who finds that animal considerations have been neglected in the discussion on sustainable development, including on the SDGs on food, water, sustainable consumption and production, conservation, and climate change. The paper argues that while the relationships between sustainable development and WE issues are highly related, these debates have evolved in a rather disconnected manner.

### SDG 1: Poverty–WE Earning and Sparing Income

**Figure 1:**
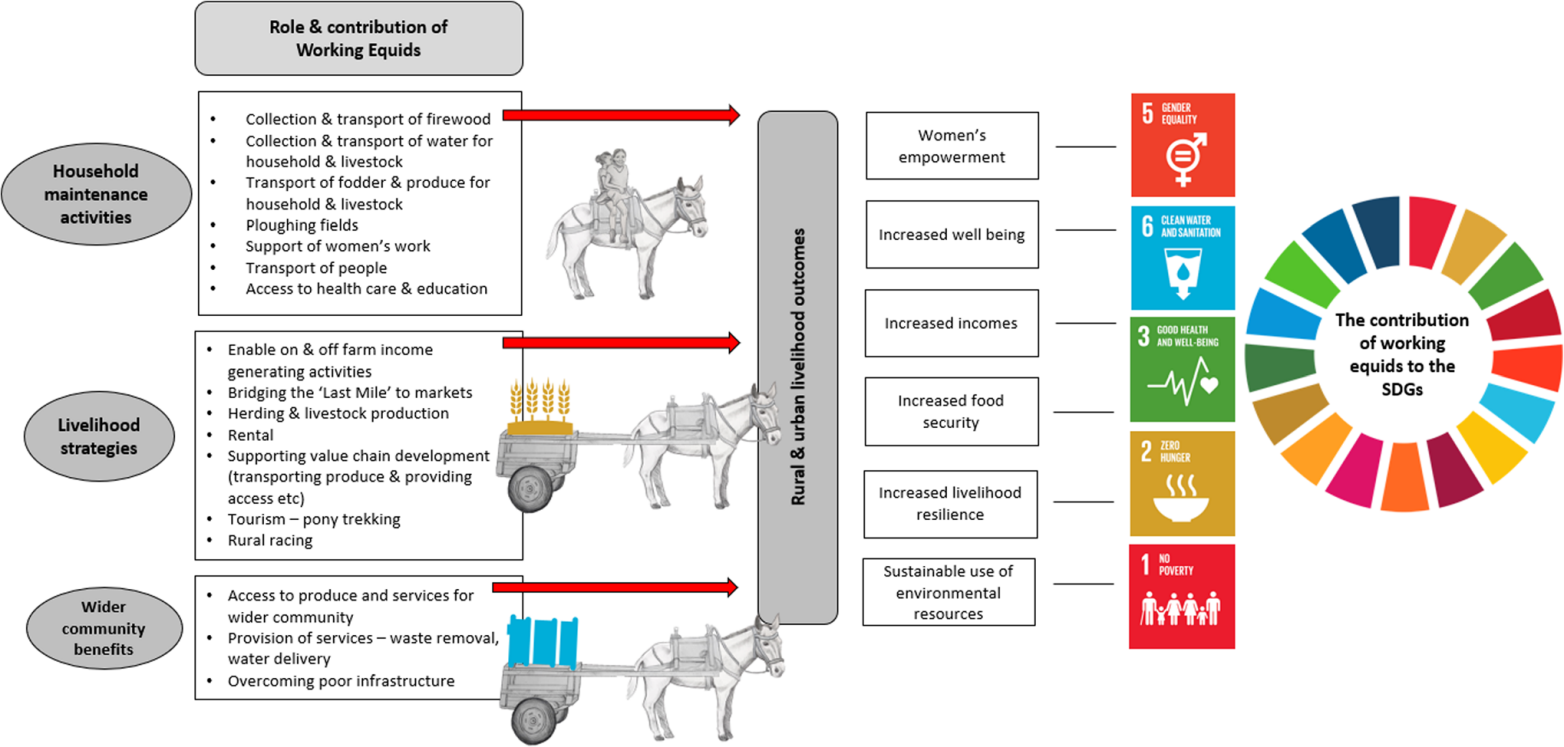
The roles and contributions of WE in rural communities and smallholder livelihoods. (Left) Specific examples of contributions by WE are shown, along with (Right) the links between these contributions and Sustainable Development Goals 1, 2, 3, 5 and 6.

WE contribute to income generation when owners earn money from them and contribute indirectly by serving as farm labour that would otherwise require cash outlay. Some donkey owners in Kenya earn 87% of their annual gross income from commercial transport (Maichomo et al. 2019). In southern Ethiopia, income generated from equine use accounted for 14% of total income across three districts (Admassu and Shiferaw 2011). Indirect, or cash substitution contributions are seen in Pakistan, where WE may support 100% of the annual income of households who rely on crop and milk sales, transporting goods across the ‘missing mile’ to markets and milk collection hubs (Valette 2015) (Fig. 1).

A recent survey found the most frequent roles of WE in LMICs were in transport of goods (25%), crops (24%) and people (18%) (Wild et al. 2021a). Crop transport was most important in Latin America, people transport in Africa, and goods transport in Asia. These activities generate income or substitute for expenditure, helping the household make ends meet.

Case studies illustrate the diversity of contributions to income generation. In Mexico 91% of equid owners derived some money from their equid, and for 22% of owners, it was their main source of income (Haddy et al. 2021). In West Africa, meanwhile, WE are used for transport in many agricultural and economic contexts, including transport of goods to and from food production sites, movement of harvested products to houses for consumption, and income generation through being hired for various works (Starkey 2011). In Kenya, initial cash injections from selling a donkey can enable owners to meet immediate financial needs like school fees; however, because of market failure, the lifetime value of working donkeys, which enable their owners to earn 11,390 Kenyan shillings (KES) per month, far exceeds the price they get for the sale (Maichomo et al. 2019). WE may be the most valuable asset that people own, and they therefore require the largest expense to replace; on the other hand, their high value means they can serve as a loan guarantee or be liquidated for emergency expenses. These financial inclusion services are especially important given equid owners are often among the poorest and most marginalised households in LMICs (Watson et al. 2020). The COVID-19 disruption has drawn attention to their importance as owners have been forced to sell WE, in an attempt to cope with loss of income and livelihood opportunities (Wild et al. 2021a). It is imperative that the contribution of WE be included in strategies to address the setbacks caused by the pandemic on the attainment of this SDG, especially in poor and marginalised households for example, restocking.

As well as moving people, crops, water, and goods, WE also contribute to income generation and zero poverty through involvement in industry and infrastructure (e.g., production of bricks and extraction of coal and minerals) in Egypt, India, Nepal, Mexico, Afghanistan, and Pakistan (Valette and Mitra 2017). WE are widely used in the transport of construction materials for housing (Kidanmariam 2000; Starkey 2004).

One novel, and of considerable concern to some, is the inter-continental value chain for “ejiao”, a gelatine produced from donkey skin and used in traditional Chinese medicine and beauty products. Africa is the epicentre of this trade, which has depleted stocks and resulted in an illegal trade of donkeys (Goodrum et al. 2022). Replacing stocks through breeding is slow, as donkeys have low fertility and long gaps between offspring (Brooke 2020a).

### SDG 2: Zero Hunger–WE in Food Value Chains

Income security is important for food and nutrition security but WE are also directly involved in food lifecycles contributing to input provision, production, transport and waste management thereby improving food security for owners and surrounding communities (Brooke 2021) (Fig. 1).

In Burkina Faso, losing a donkey translates to a at least 50% loss in most cultivated products, including 89% of cowpeas (Brooke 2018). Conversely, in parts of Senegal, meanwhile owning a donkey is associated with 78% more groundnuts, 46% more maize and 45% more millet produced (Brooke 2018). WE also support production of livestock by carrying water and fodder. In the groundnut basin and the sylvo-pastoral zone in Senegal, donkeys supply water to 400,000 small ruminants every day (Brooke 2018). Elsewhere in Africa, a study in the Amhara Zone of Ethiopia found that horses were used for ploughing fields (Asmare and Yayeh 2017). In a region in India, meanwhile, equid-associated income generation enabled the purchase of almost 80% of annual human food requirements (Brooke 2021).

Equines also make an important input into growing and transporting sugar cane, which accounts for 80% of global sugar production. Six of the ten biggest producers of sugar cane are LMICs (including Brazil, India, China and Thailand) (FAOSTAT 2022), and production relies on the labour of people and animals. For example, transport of cane stalks by mules and horses is still the dominant system for small-scale producers in Colombia, while in the Dominican Republic, ox carts predominate (Starkey 2011). Similarly, in Guatemala, 85% of coffee is transported by WE (Chang et al. 2010; Mosquera and Álvarez 2019). WE also transport firewood and water for household use, which are both vital enablers of food security for households (Wild et al. 2021b).

For crop cultivation, equid manure has long been valued as a fertiliser: it is richer in nitrogen than cow manure, and because of the large amount of organic matter is a good soil conditioner, and substrate for vermicosting and mulch. For example, in central Kenya, manure from donkeys was often used as fertilizer for farmed crops such as rice (Gichure et al. 2020).

### SDG 3: Good Health and Well-being—WE Facilitating Access to Health Care

Where distance or terrain are barriers to accessing healthcare services, WE may be the only available form of transport (particularly for people who are older, pregnant, impaired, or ill). In Ethiopia and West Africa, for instance, they are used to transport patients to clinics and nurseries (Admassu and Shiferaw 2011; International Coalition for Working Equids 2019). One Tunisian survey reported that 90% of respondents use income generated by working animals to afford medical treatments (International Coalition for Working Equids 2019). The access to water afforded by WE also improves health. In Lesotho, World Horse Welfare (2019) works with a health non-governmental organisation (NGO) that provides logistical support to the Ministry of Health and uses WE in inaccessible mountainous regions to transport diagnostic samples from health posts to laboratories. Approximately 5,700 blood samples (e.g., for HIV or COVID-19 testing) are transported annually by these horse riders (Unpublished project monitoring data, Riders for Health Monthly Monitoring Data 2019). Increased access to accurate and timely results has a critical impact on individual and community health in these remote highland areas.

Although not specifically mentioned in SDG 3, One Health is of growing importance as an approach for optimising health of people, animals, and ecosystems (Dye 2022). Improvements in animal health and welfare, including strengthened animal health systems, benefits global health security by reducing zoonotic disease transmission (Horsetalk 2020; Lönker et al. 2020). While case reports of zoonotic infections directly from horses remain low, there is a high potential for underreporting due to lack of knowledge among health professionals (Sack et al. 2020). Globally some 56 diseases are recorded as transmittable from equines to man (Sack et al. 2020). Some of these are emerging infectious diseases and thus may be less known to both the equine and human medical community; they may pose a risk both for WE and their handlers.

The “one welfare” concept (Garcia Pinillos et al. 2016), related to and derived from the One Health movement, views the health or welfare of humans, ecosystems and the health and welfare of individual animals as one. It provides a framework to link animal and human health to sustainability of the environment and may be used to emphasize the role of WE.

### SDG 5: Gender Equality–WE Helping Women

Women across the world depend on WE to support them with time-consuming tasks such as transporting water, firewood, building materials, and fodder (Starkey 2011) – in Ethiopia, for example, 40% of surveyed households reported that donkeys helped to reduce women’s work burden (Admassu and Shiferaw 2011). Income from working animals helps women pay for household essentials and can be a factor in gender empowerment. Women are frequently the caregivers for WE (Vasanthakumar et al. 2021).

### SDG 6: Clean Water and Sanitation–WE Transporting Water

Millions of people worldwide, mainly women and girls, spend up to 200 million person-hours each day collecting clean water (Farley 2018). WE are critical for providing access to water for communities and for all food-producing animals and agriculture. In Africa, water transport is the second most important function of WE (Wild et al. 2021a). Studies of water use must consider the critical impact that WE have in enabling communities to access water. In Tunisia, 80% of survey respondents in remote and mountainous regions relied on their donkeys or mules to access and carry fresh water (International Coalition for Working Equids 2019) In the Mauritanian city of Nouakchott, meanwhile, water carriers use donkeys to carry 400 L of water at a time, supplying the majority of households and businesses (International Coalition for Working Equids 2019). WE also play a role in sanitation contributing to clean water. In Karachi, for instance, waste collection was the primary source of income for 89% of donkey owners (Shah et al. 2019).

While WE are widely agreed to make substantial contributions to six SDGs (SDG 1, SDG 2, SDG 3, SDG 5 and SDG 6), they also support other SDGs to a lesser extent or to an extent that has been less examined. Contributions to SDG 1 and 2 have co-benefits to SDG 8 (decent work) and SDG 10 (reduced equality). WE provision of fertiliser and energy contributes to reduced greenhouse gases (SDG 13) and affordable, clean energy (SDG 5). WE can enhance the attractiveness and liveability of urban and rural and habitats (SDG 11 and 15).

### The Background to the Neglect of WE

Given the importance of WE and their welfare in LMICs and their documented benefits to livelihoods, the lack of prominence of WE and equid welfare in national policy and agricultural development is surprising and, in this section, we review neglect at the levels of government, national authority, civil society and smallholder owners and users.

### The Background to the Neglect of WE—Results

*Neglected owners* Most WE are kept by smallholder farmers or pastoralists, often from marginalised communities (van Dijk et al. 2014) Governments, commonly believing that agricultural modernisation and farm consolidation are the agriculture of the future, do not always prioritise these sectors (and therefore do not make resources available for WE). There is a perception that use of WE is a retrograde step in agricultural development.

*Missing measures* WE are regarded by policymakers as less valuable than ruminants. Compared to other livestock, there is a relative lack of evidence collected on WE economic contributions (Zaman et al. 2014); this subsequently results in their exclusion from initiatives, policies, and research (Soulsby et al. 2004; Stringer et al. 2015). For evidence-based decision-making, regular collation of the socio-economic contribution of WE, and the burden of poor animal welfare and disease, is required (Brooke 2020b). This is a complicated task, as WE’ contributions are difficult to standardise and are often not monetised. For example, it is easier to assess the amount and value of milk produced in a district than it is to assess the value of all the various work performed by WE in bringing children to school, transporting water from wells and products to markets, etc. Even monetised on- and off-farm income-generating activities, such as renting WE for draught, transporting goods and accessing services, are poorly researched and quantified.

*Atypical livestock* Public officials commonly think of livestock in terms of animal source food and animal by-products. WE’ economic contributions are seen as indirect and are therefore often missed. A project in Panama, for example, found that WE were a vital part of the process of raising cattle (herding, carrying supplementary food, monitoring), but this was not recognized by governmental officials (Pile et al. 2021). In the Lesotho highlands, horses are regarded as essential for herding ruminants and transporting wool and mohair to market (Unpublished project Baseline Focus Group Discussions with Equid Owners, Mafeteng District 2018).

*Donor neglect* LMIC governments are strongly influenced by donor priorities. Many development priorities such as gender equality, climate change and antimicrobial resistance were not spontaneously embraced by LMICs but were instead the results of donor interest and funding. Donors rarely prioritise WE or include them in requests or discussions on policies and resource allocation. International donors generally do not consider WE in either the livestock or livelihood categories, often focusing on broad strategies (e.g., poverty reduction) rather than specific tactics (e.g., donkeys as a pathway out of poverty) (Geiger et al. 2020a). For the issues which donors care about (e.g., no poverty and zero hunger), they are not aware of the contribution of WE. A particular challenge is that WE are not linked to contributions to the SDGs, which are currently the main international development agenda (International Coalition for Working Equids 2019).

*Policies and processes that are not equid-inclusive* In some countries, WE are not formally defined as “livestock” in government policies or legislation, making it difficult to ensure that laws apply to WE equally (Babayani 2019). This loophole has frequently been exploited by people in the donkey skin trade. Because donkeys are not specifically listed in legislation, no permits or regulations legally apply to their movement or slaughter, and they can thus be exploited without legal repercussion) (Babayani 2019).

Old and outdated animal welfare legislation reinforces the low status of WE. For example, Lesotho has an outdated basic anti-cruelty law, and there appears to be no proactive animal welfare enforcement in the country (World Organization for Animal Health 2017). While an animal welfare policy was initiated in 2008, it is yet to be approved and brought into legislation. Additionally, in World Organisation for Animal Health (WOAH) animal slaughter recommendations, donkeys are not included (World Organisation for Animal Health 2017).

In Zimbabwe, Ministry of Agriculture policies do not appear to prioritize WE. Resource allocation in the livestock sector is focused on cattle production, with lesser emphasis on sheep, goats and poultry. One interviewed official noted that “the government is silent on the issue of WE in this country. Donkeys are taken for granted, they are not included, there are no strategies or programmes to deal with them. Beef is the priority’’(Unpublished Project Report World Horse Welfare 2016). Zimbabwe has a Prevention of Cruelty to Animals Act (1986) and a Scientific Experiments on Animals Act (1963), but this legislation only includes basic provisions regarding animal cruelty and abandonment. The country does not have an explicit animal welfare policy or strategy (World Organization for Animal Health 2017).

*Lack of equid expertise* Training on WE at agriculture colleges, veterinary schools and universities is limited. In a survey of veterinarians working for Brooke, 50% reported little-to-no equine veterinary education prior to joining the organisation (Hirson et al. 2014). Therefore, even if veterinary services are made available by LMIC governments, they often cannot deal with equid-centric issues. For example, in Cambodia, there were only two veterinarians who had the knowledge and confidence to work with equine species (Rogers 2014). Most government district veterinary officers do not have the specialised knowledge or expertise required to handle WE (Brooke 2019a), and they report a lack of confidence in addressing equine health cases. Agriculture extension workers report having very little practical experience with WE, and farmers therefore rarely approach them for assistance. In South Africa, state veterinarians who work in rural communities with subsistence farmers do not stock medication and vaccines for WE and thus seldom treat these animals (Ward, P., personal communication. 2021).

Owners rarely have access to training and extension services that include WE. They may have kept WE for generations and have a good understanding of their animals, or they may have recently acquired a horse or donkey and have no previous experience. When owners seek support with WE, NGOs, extension workers and state veterinarians may not have the required expertise or the remit, as WE are not a development priority.

It must also be recognised that many equid owners have good knowledge and skills but may be limited by access to financial resources, time, animal health services and water – they may therefore not be motivated to adopt practices that improve animal welfare. We recommend an approach that recognises that capability (knowledge and skills), opportunity (access to resources) and motivation are all required for human behaviours to change (West and Michie 2020). Adopting this approach will ensure that governments and NGOs work with people to identify the priorities for their animals and will give them the agency and support to seek sustainable and context-appropriate local solutions (Valette 2014).

*Uncounted WE* Another manifestation of equid neglect is the lack of accurate data on their populations and functions. The FAO Corporate Statistical Database (FAOSTAT) is the most reliable source; it likely underestimates numbers, however, because WE are regarded as less economically important than other livestock (Norris et al. 2021). According to FAOSTAT, there were 123 million total WE (comprising 55 million donkeys, 60 million horses and 8 million mules) in 2019 (FAOSTAT 2019). Assuming that WE constitute 85% of the world’s equid population (Burn et al. 2010), FAOSTAT implies that in 2019 (or the last year of reporting, if not 2019), there were 101 million WE.

The two countries with the most WE are Mexico (13 million) and Ethiopia (11 million). Mexico also has the most mules and Ethiopia the most donkeys. Mongolia has the most WE per thousand people (1,330), followed by Iceland (776) and Sudan (201). Africa is the region with the most WE overall, followed by Asia. These estimates correspond with those of Allan (2021), which used slightly different definitions. A recent study based on FAOSTAT found the number of donkeys is globally increasing at a rate of ~ 1% per annum, with the largest increases in Africa (Norris et al. 2021), whilst mule populations are in decline at a rate of ~ 2% per annum. In some countries, WE are not included in livestock censuses or in reporting by government extension agents. In Zimbabwe, donkey population statistics have not been included in the Ministry of Agriculture’s annual livestock survey reports since 2017. In South Africa, the most recent livestock census was conducted in 2018, but the official report only includes statistics for ruminants, pigs and chickens; no mention is made of WE. In contrast, in Lesotho, horses and donkeys are included in the livestock census. However, the last survey was conducted in 2014. Official annual estimates of equid populations are based on these figures.

Information on horse and donkey populations in rural communities is not collected systematically by governments, and current figures are often based on estimates made over a decade ago. The situation is similar for data on camelid numbers, a question often asked but rarely reliably answered – the best guess is around 40 million (Faye 2020). “Numbers” data are important for, among other factors, understanding the dynamic population shifts and massive cross-border movement of animals resulting from the legal and illegal donkey skin trades.

*Attitude towards WE* In some cases, owners have a negative attitude towards WE (Geiger et al. 2020; Luna et al. 2017). For example, in Costa Rica, WE are sometimes viewed as tools/machines and are used for fulfilling work rather than considered as sentient beings. Some owners would rather have a motorcycle or a vehicle than an equid if given the choice. Nonetheless, there are many examples of compassion for WE, and they are often considered part of the family. For example, children in Mexico said “grooming and love” were needed by donkeys (Tadich et al. 2016).

*Equid exclusion* Equid markets are often absent or underdeveloped. For example, in Zimbabwe, there is no formal market for donkeys, and sales occur only through private transactions (spot markets). In contrast, large monthly ruminant markets are regularly held at various locations across the country (Unpublished Project Report; Veterinarians for Animal Welfare in Zimbabwe & World Horse Welfare 2020). There are exceptions, such as extensive equid market systems across Asia where WE are bought and sold for working in brick kilns. Hundreds of ‘equine fairs’ are also held across India, where thousands of animals are brought together for ‘markets’ that last several days and horses may be offered for sale in north African and Sahelian ‘horse festivals’.

*Exploitable WE* Willing WE continue to work when they are poorly treated, removing an incentive for humane treatment. For example, in a study of 227 working horses in India and Pakistan, all horses were lame ( Pritchard et al. 2005). Unsurprisingly, studies have shown that improving equid welfare increases productivity, an underused incentive for humane treatment (Davis and Harness Aid 2008; SPANA 2020). Cultural differences in animal-related values and beliefs are complex and still not fully understood. We need a greater understanding of the complex livelihood systems in which WE are involved.

In addition to these major drivers, literature and the authors’ experience suggest additional constraints, including the impairment of equid productivity by poor health, husbandry and feeding and the lack of appropriate policies to safeguard WE contributions.

### Factors Reducing the Current and Potential Contribution of WE to SDGs

Given the important contributions of WE to the SDGs, it is also important to examine the factors that hinder this, and the barriers that, if addressed, could lead to still greater contributions.

The drivers of equid neglect identified by animal welfare experts in our expert integrative review can be conceptualised as constraints to WE ability to contribute to the SDGs. These are summarised in Table 1, along with potential solutions.

**Table 1:** Constraints on Equid Contributions to SDGs and Potential Solutions.

Level	Barrier to contributing to SDGs	Potential solutions
Government	WE in neglected farming systems	Evidence generation and advocacy on the importance of smallholder and pastoralist systems and the role of WE
	Donors do not influence LMICs to prioritize WE	Demonstrate how WE contribute to the SDGs
	WE are not considered typical livestock	Better integration of equid-relevant information into reporting
	Lack of equid expertise	Tertiary level training; raising awareness
	Lack of information on equid populations and contributions	Generation of better equid censuses and data on contributions to commodity value chains
	Lack of appropriate policies to safeguard equid resources – especially in the wake of the COVID-19 pandemic	Policy analyses and recommendations highlighting the impact of failure to safeguard WE; international and national guidelines
Community	Lack of equid expertise	Vocational training; raising awareness
	Prejudices and misperceptions around WE	Studies on the drivers of misperceptions and the means to redress them; empathy training; animal welfare school clubs
	Lack of functioning markets for WE and equid products	Market development; digital market information systems
	Lack of access to equid health, husbandry and feed inputs	Strengthening of existing animal health systems; deployment of a Human Behaviour Change approach to identify community priorities and work together to seek solutions

The suggested solutions occur in a context of external factors both hostile and propitious to equid development. For example, there is continuing support for mechanisation of agriculture (despite its environmental costs) and massive road construction initiatives favour motorisation. However, new roads will not reach deep into rural areas; therefore, local transport on poor or no roads will still be needed (Burridge et al. 2021). In focus groups with community stakeholders in Colombia where discussions were held on government initiatives to replace WE with motorised transport, three themes were identified: ‘culture’, ‘practicality’ and ‘profitability’. Individuals living in one municipality were concerned because of culture reasons. Elsewhere, there were concerns about practicality and profitability of this scheme, including (a) terrain that is impassable by motor vehicles, and (b) the negative effect on personal economy in other areas of Colombia where local governments had already replaced WE with motorised vehicles.

Moreover, the increasing price of fuel and efforts to reduce greenhouse gases may encourage more use of WE. Indeed, climate change may open areas previously unsuited to WE. For example, climate change is reducing the distribution of tsetse in parts of Africa, making it possible to keep horses there (Longbottom et al. 2020). Climate change may lead to other ecosystems becoming more arid and thus more suited to WE. Since the start of equid population reporting by the FAO in the 1960s, there has been a continuous increase in the number of WE in LMICs (Allan 2021) – there is little reason to think this will be reversed.

#### Future Opportunities and the Way Forward

Among the many possible actions identified in this paper, we emphasise the following key opportunities for enhancing the appreciation of, and contributions by, WE include:

- Better quantification of the number and economic contributions of WE, particularly in relation to commodity value chains. This will be facilitated by individual registration of WE.
- Greater technical alignment and integration with international agriculture, environmental impact, and climate change. This can be supported by intentional incorporation of WE in One Health capacity building and operationalisation.
- Enhancement of national and regional human resource capacity, leadership and coordination in LMICs. This can fostered through better inclusion of WE in primary, secondary, tertiary and vocational education and by advocacy for WE.

The framework we have proposed organizes WE contributions according to SDGs. As SDGs are now the guiding principle for development investments, this categorisation can help planners see how greater attention to WE can help them attain their goals. The framework takes a value chain lens which helps differentiate the contributions by WE and identify constraints and opportunities in different contexts.

## Conclusions

This paper discusses the benefits that WE provide in LMICs, their contributions to the SDGs, and importantly, the many barriers they face in acquiring proper recognition as the livestock that they are. We have also discussed the associated lack of policy and the extension and development initiatives that should be attributed to WE.

This review has highlighted WE contributions to the SDGs and the failure of many countries and organisations to realise the benefits for their communities by meeting the many areas of the needs of WE and their owners, to help motivate changing policies, systems and attitudes relating to WE.

The establishment of an effective dialogue between policy makers and stakeholders is necessary to agree conversation and actions to introduce legal, policy and fiscal measures to implement changes that will positively impact WE and their owners. Key areas to be addressed include improved enumeration and alignment within the agricultural and environmental agenda, as well as strengthened capacity. Better recognition of WE’ contributions would benefit the health and welfare of both WE and their owners, whose livelihoods so often depend upon these “invisible” animals.
